# MicroRNA expression profiles identify disease-specific alterations in systemic lupus erythematosus and primary Sjögren's syndrome

**DOI:** 10.1371/journal.pone.0174585

**Published:** 2017-03-24

**Authors:** Ji-Qing Chen, Gábor Papp, Szilárd Póliska, Krisztina Szabó, Tünde Tarr, Bálint László Bálint, Péter Szodoray, Margit Zeher

**Affiliations:** 1 Division of Clinical Immunology, Faculty of Medicine, University of Debrecen, Debrecen, Hungary; 2 Genomic Medicine and Bioinformatic Core Facility, Department of Biochemistry and Molecular Biology, Faculty of Medicine, University of Debrecen, Debrecen, Hungary; 3 Centre for Immune Regulation and Department of Immunology, University of Oslo, Oslo University Hospital, Rikshospitalet, Oslo, Norway; Instituto Nacional de Ciencias Medicas y Nutricion Salvador Zubiran, MEXICO

## Abstract

The discovery of microRNAs (miRNAs) and their critical role in genetic control opened new avenues in understanding of various biological processes including immune cell lineage commitment, differentiation, proliferation and apoptosis. However, a given miRNA may have hundreds of different mRNA targets and a target might be regulated by multiple miRNAs, thus the characterisation of dysregulated miRNA expression profiles could give a better insight into the development of immunological disturbances in autoimmune diseases. The aim of our study was to examine the changes in miRNA expression profiles in patients with systemic lupus erythematosus (SLE) and primary Sjögren's syndrome (pSS). Eight SLE patients, 8 pSS patients and 7 healthy subjects were enrolled in the investigation. MiRNAs were isolated from peripheral blood mononuclear cells, and expression patterns were determined with Illumina next-generation sequencing technology. Since the immunopathogenesis of pSS and SLE encompasses pronounced B cell hyperactivity along with specific autoantibody production, we paid a special attention on the association between miRNA expression levels and altered peripheral B cell distribution. In SLE patients 135, while in pSS patients 26 miRNAs showed altered expression. Interestingly, the 25 miRNAs including miR-146a, miR-16 and miR-21, which were over-expressed in pSS patients, were found to be elevated in SLE group, as well. On the contrary, we observed the down-regulation of miR-150-5p, which is a novel and unique finding in pSS. Levels of several miRNAs over-expressed in SLE, were not changed in pSS, such as miR-148a-3p, miR-152, miR-155, miR-223, miR-224, miR-326 and miR-342. Expression levels of miR-223-5p, miR-150-5p, miR-155-5p and miR-342-3p, which miRNAs are potentially linked to B cell functions, showed associations with the B cell proportions within peripheral blood mononuclear cells. The observed differences in miRNA expression profiles and the better understanding of immune regulatory mechanisms of miRNAs may help to elucidate the pathogenesis of SLE and pSS.

## Introduction

The discovery of microRNAs (miRNAs) and their critical role in genetic control opened new avenues in understanding of the intricate interplay of inherited and acquired factors leading to disease development. MiRNAs are single-stranded, endogenous non-coding RNAs, ranging from 18 to 25 nucleotides in length [1,2]. After the first study revealing the posttranscriptional regulatory role of non-coding RNAs on gene expression in Caenorhabditis elegans [3], small RNAs with similar functions were identified in other animal models, such as Drosophila and zebrafish [4]. Since then, several studies on miRNAs were reported in both animal models and humans, which shed light on their biology and mechanism of action. It is already known that mature miRNAs interact with specific messenger (m)RNAs to regulate gene expression. Target mRNA is recognized by the 2–7 nucleotides of the so called ‘seed’ region of miRNAs [5]. When the complementary base pairing is perfect or near-perfect, endonucleotic cleavage is induced, which leads to the degradation of mRNAs. When the base pairing is incomplete, the formation of double-stranded RNAs resulting from the binding of miRNAs, leads to translational repression [6,7]. MiRNAs regulate approximately 90% of protein-coding genes, and play a central role in various biological processes including immune cell lineage commitment, differentiation, proliferation, apoptosis and maintenance of immune homeostasis [8]. Alterations in miRNA regulation seem to be highly related to the development of immune dysfunctions and autoimmunity. In the last years, changes in miRNA expression have been identified in certain autoimmune diseases including rheumatoid arthritis (RA), systemic lupus erythematosus (SLE) and primary Sjögren’s syndrome (pSS), as well [9,10]. Consequently, these molecules may be regarded as novel and attractive biomarkers specific for different autoimmune disorders; moreover, it has been also suggested that miRNA-targeting treatment might be more selective than the other therapeutic regiments in autoimmune diseases [11]. However, it has to be taken into consideration that a given miRNA may have hundreds of different mRNA targets, and a given target might be regulated by multiple miRNAs, thus, the intricate interplay between specific miRNAs and the functionally targeted genes is not elucidated yet. Additionally, genome-wide surveys identified many single nucleotide polymorphisms (SNPs) in the predicted miRNA target sites, as well as in miRNAs themselves [11]. In some instances, SNPs have been shown to alter miRNA function, thus possibly contributing to disease development. The better understanding of the immune regulatory mechanisms of miRNAs by pathway-based exploratory analyses and the mapping and characterization of miRNA SNPs may help not only to elucidate the pathogenesis of autoimmune conditions but also can lead to the development of complex therapeutic approaches in patients with immunological disorders.

Systemic lupus erythematosus is a clinically heterogeneous, chronic systemic autoimmune disease characterized by the presence of autoantibodies directed against nuclear antigens and damage of multiple organ systems, including renal, cardiovascular, musculoskeletal neural and cutaneous systems. SLE is a relapsing and remitting disease, encompassing mild to moderate forms, and also severe, progressive variants with a potentially debilitating, even fatal outcome [12]. Primary Sjögren’s syndrome is a common systemic autoimmune disease that affects primarily the exocrine glands, leading to decreased lachrymal and salivary secretion. Besides the characteristic glandular symptoms, other systemic symptoms, denoted as extraglandular manifestations (EGM), can also be found in a subset of patients [13]. The immunopathogenesis of both pSS and SLE are not fully elucidated yet; albeit enhanced B cell activity and pronounced autoantibody production are the hallmark of these diseases.

The aim of our study was to examine miRNA expression profiles in patients with pSS and SLE in order to remark the associations between the dysregulated miRNAs’ expression and the development of the diseases. Since the immunopathogenesis of pSS and SLE characterised by pronounced B cell hyperactivity along with specific autoantibody production, we paid a special attention on the association between miRNA expression levels and altered peripheral B cell distribution. Additionally, contrary to microarray analysis, the RNA sequencing technique gave us an opportunity to detect and recognise miRNAs with SNPs, as well.

## Materials and methods

### Patients and healthy individuals

Eight female patients with pSS (mean age: 55.12 ± 7.49 years, range from 42 to 64 years) and 8 female patients with SLE (mean age: 45.88 ± 10.58 years, range from 36 to 66 years) were enrolled in the study. All patients were recruited from the Outpatient Clinic for systemic autoimmune diseases at the Division of Clinical Immunology, University of Debrecen, where they received regular follow-up treatment. The recruitment of participants was between 14-12-2015 and 29-02-2016. The average disease duration was 13.40 ± 7.94 years in pSS, while 14.14 ± 9.14 years in case of SLE. The diagnosis of pSS was based on the European-American consensus criteria [14]. Among pSS patients, 4 suffered from EGMs, while 4 had only glandular symptoms. The distribution of EGMs of pSS patients were as follows: polyarthritis n = 4, Raynaud’s phenomenon n = 3, vasculitis n = 1. The exclusion criteria included therapy with immunosuppressive/immunomodulatory agents. Vasculitis or other EGMs needing immunosuppressive treatment were newly recognised. Patients with SLE fulfilled the corresponding diagnostic criteria for lupus [15,16]. All of the SLE patients received per os methylprednisolone therapy with an average dose of 4 mg daily; the dose of the treatment did not exceed 8 mg methylprednisolone per day in any case of SLE patients. The blood samples were collected from SLE patients 24 hours after taking the regular methylprednisolone medication. We assessed the actual disease activity of SLE patients and Systemic Lupus Erythematosus Disease Activity Index (SLEDAI) scores were calculated. None of the lupus patients showed clinical activity or had SLEDAI score higher than 4. The control group consisted of seven age-matched (mean age: 49.78 ± 7.31 years, range from 39 to 61 years) healthy female volunteers. No patients or controls enrolled in this study had ongoing infections, either viral or bacterial. Table 1 summarizes the demographic and clinical data of the individuals included in the study. Informed written consent was obtained from all subjects enrolled in the investigation, and the study has been approved by the Ethics Committee of our University and the Policy Administration Services of Public Health of the Government Office (protocol number: IF-13052-9/2015). All experiments carried out were in compliance with the Declaration of Helsinki.

**Table 1:** The demographic and clinical data of study individuals.

	pSS patients	SLE patients	Healthy controls
**Number**	8	8	7
**Age in years, mean ± SD [range]**	55.12 ± 7.49 [42–64]	45.88 ± 10.58 [36–66]	49.78 ± 7.31 [39–61]
**Gender, male/female**	0/8	0/8	0/7
**Disease duration in years mean ± SD [range]**	13.40 ± 7.94 [3–22]	14.14 ± 9.14 [2–32]	n.a.
**Extraglandular involvement, n (%)**	4 (50)	n.a.	n.a.
**SLEDAI score**	n.a	1.75 ± 1.67 [0–4]	n.a.

pSS, primary Sjögren's syndrome; SLE, systemic lupus erythematosus; SD, standard deviation; SLEDAI, systemic lupus erythematosus disease activity index

### Sample handling

Peripheral blood samples obtained from each study subjects were collected and mononuclear leukocytes (PBMCs) were separated by Ficoll-Histopaque (Sigma-Aldrich, St Louis, MO, USA) density-gradient centrifugation.

### RNA isolation

RNA samples were isolated from PBMCs using Trizol reagent (MRC, Cincinnati, Ohio, USA) according to manufacturer’s protocol. RNA quantity was measured by UV photometry using NanoDrop instrument (Themo Fisher Scientific, Waltham, MA, USA). RNA quality was checked by Agilent BioAnalyzer and RNA samples with RIN > 7 were used for the further applications.

### RNA sequencing (small RNA-Seq) library preparation

Sequencing libraries for small RNA-Seq were generated from 1 μg total RNA using TruSeq Small RNA Sample Preparation Kit (Illumina, San Diego, CA, USA) according to the manufacturer’s protocol. Fragment size distribution and molarity of libraries were checked on Agilent BioAnalyzer DNA1000 chip (Agilent Technologies, Santa Clara, CA, USA). Subsequently single read 50 bp sequencing run was performed on Illumina HiScan SQ instrument (Illumina, San Diego, CA, USA).

### Determination of B cell subsets

For the identification of different lymphocyte subpopulations, PBMCs were stained then assessed using flow cytometer, as described previously [17]. For identification of B cell subsets, we used the combination of IgD-fluorescein isothiocyanate (FITC)/CD27-phycoerythrin (PE)/CD19-phycoerythrin-Cyanine dye 5 (PE-Cy5) (Beckman Coulter Inc, Fullerton, CA, USA and Immunotech, Marseille, France) and CD38-FITC/CD27-PE/CD19-PE-Cy5/CD24-allophycocyanin (APC) (BD Biosciences, San Diego, CA, USA and Beckmann Coulter and BioLegend, San Diego, CA, USA). According to the expression of IgD, CD27, CD38 and CD24 cell surface markers, the following B cell subsets were identified: CD19^+^IgD^+^CD27^-^ naive B cells, CD19^+^IgD^+^CD27^+^ non-switched IgM memory B cells, CD19^+^IgD^-^CD27^+^ switched memory B cells, CD19^+^IgD^-^CD27^-^ double negative (DN) B cells, CD19^+^CD38^-^CD24^hi^CD27^+^ primarily memory B cells, CD19^+^CD38^hi^CD24^hi^CD27^-^ transitional B cells, CD19^+^CD38^+^CD24^+^ mature-naive B cells and CD19^+^CD38^hi^CD27^hi^ plasmablasts. Cells were quantified as their percentage in the CD19^+^ lymphocyte population. Fluorescence Minus One controls were used in all procedures. The stained cells were measured by FACS Calibur flow cytometer (Becton Dickinson, Franklin Lakes, NJ, USA) and data was analysed using FlowJo Software (Treestar, Ashland, OR, USA).

### Data analysis

CASAVA software was used for pass filtering and demultiplexing process. Sequenced reads were aligned to Human Genome v19 and bam files were generated. Further statistical analyses were executed using StrandNGS software (Agilent Technologies, Santa Clara, CA, USA). Relative small RNA expression levels were calculated using DESeq algorithm. To find differentially expressed small RNAs between clinical conditions ANOVA analysis with Tukey post hoc test was performed.

To assess the distribution of the data Shapiro-Wilk normality test and Kolmogorov-Smirnov test were used. The correlations between two variables were evaluated with Pearson's correlation coefficient, while in cases of non-normal distribution, Spearman's test was used. Differences were considered statistically significant at p < 0.05.

## Results

We carried out analysis of variance (ANOVA) together with Tukey’s honest significant difference (HSD) test to evaluate the different expression patterns of miRNAs. We observed significant differences in the following cases: 25 different miRNAs were up-regulated and one miRNA was down-regulated in pSS patients compared to controls, while 135 miRNAs were over-expressed in SLE patients compared to healthy individuals.

**Fig 1:**
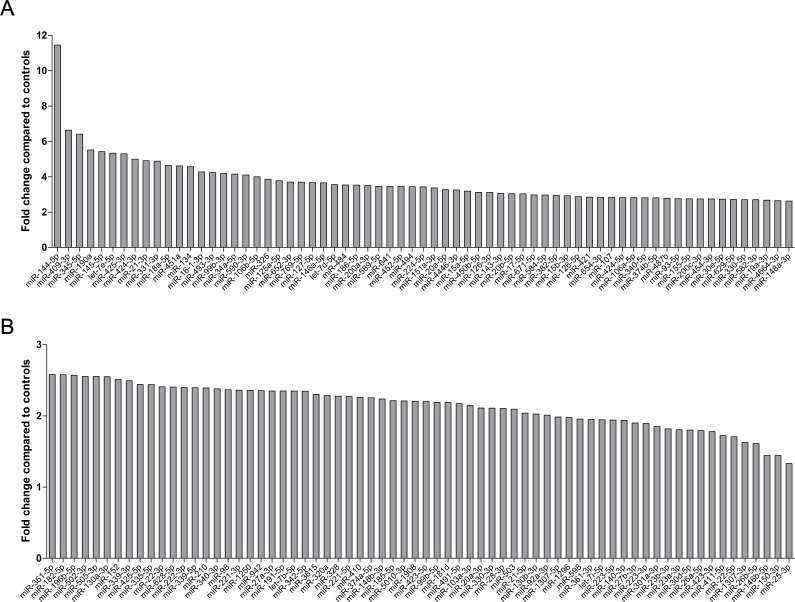
Fold changes of microRNAs in SLE patients compared to controls. MicroRNAs (miRNAs) were analysed with Illumina next-generation sequencing technology in SLE patients compared to controls. A total of 135 miRNAs were over-expressed and 113 miRNAs of them showed more than 2-fold difference. Particularly, miR-144-5p, let-7e-5p, miR-145-5p, miR-190a, miR-345-5p, miR-409-3p and miR-425-3p show more than 5-fold change in patient group compared to healthy controls.

Among all 135 significantly up-regulated miRNAs in SLE patients, 113 were over 2 fold change compared to controls. From those, let-7e-5p, miR-144-5p (11 fold change), miR-145-5p, miR-190a, miR-345-5p, miR-409-3p and miR-425-3p shown more than 5 fold change in patients group compared to healthy controls (Fig 1A and 1B).

**Fig 2:**
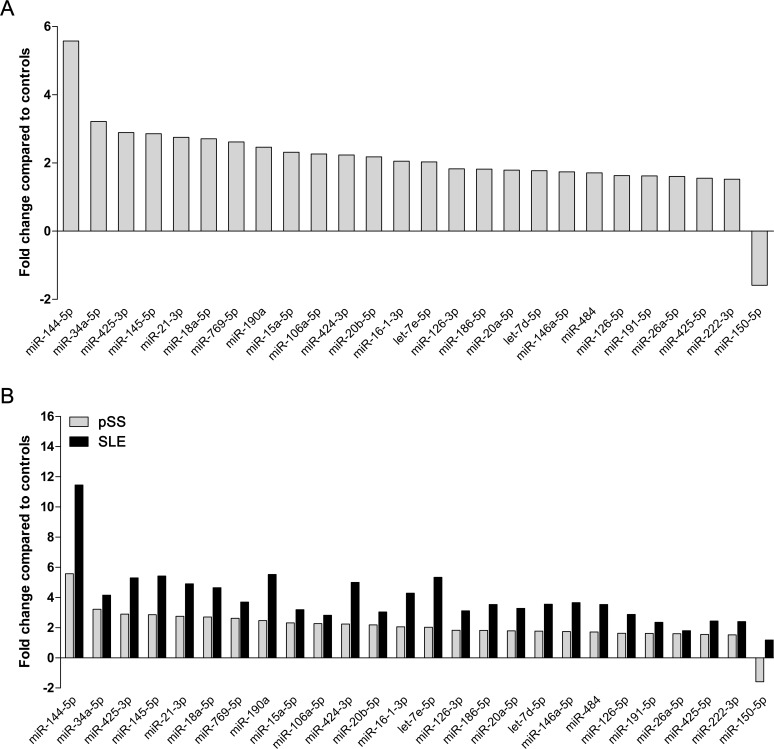
Fold changes of microRNAs in pSS and SLE patients compared to controls. (A) A total of 26 microRNAs (miRNAs) showed altered expression levels in patients with pSS compared to controls; among them, 14 miRNAs displayed more than 2-fold change. Expression of miR-150-5p was down-regulated in pSS. (B) All of these miRNAs were also analysed in patients with SLE and found to be elevated as well.

In pSS group, 25 miRNAs were significantly over-expressed in pSS compared to controls. Fourteen of them, namely, let-7e-5p, miR-16-1-3p, miR-20b-5p, miR-424-3p, miR-106a-5p, miR-15a-5p, miR-190a, miR-769-5p, miR-18a-5p, miR-21-3p, miR-145-5p, miR-425-3p, miR-34a-5p, miR-144-5p showed more than 2 fold change. Of note, all of these miRNAs were found to be elevated in the SLE group, as well. On the contrary, miR-150-5p was significantly down-regulated in pSS (Fig 2A and 2B).

Interestingly, when we compared the miRNA profiles between the subset of pSS patients with glandular symptoms only and pSS patients with EGMs, we did not find any difference in miR-150-5p expression between the subgroups of pSS patients. On the contrary, we identified the over-expression of miR-148a in patients with EGMs, which miRNA did not show any change in the whole group of pSS patients, compared to the healthy individuals.

**Fig 3:**
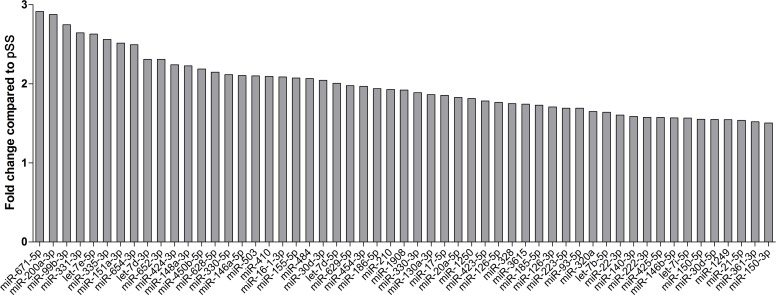
Fold changes of microRNAs in SLE patients compared to pSS patients. Fifty-five microRNAs (miRNAs) were significantly up-regulated in SLE patients compared to pSS patients. At least half of them have more than 2-fold change.

Finally, we also carried out the statistical comparison between the miRNA patterns of the SLE and pSS patients. We have found that 55 miRNAs significantly up-regulated in SLE compared to pSS. More than half of those have more than 2 fold change (Fig 3).

**Fig 4:**
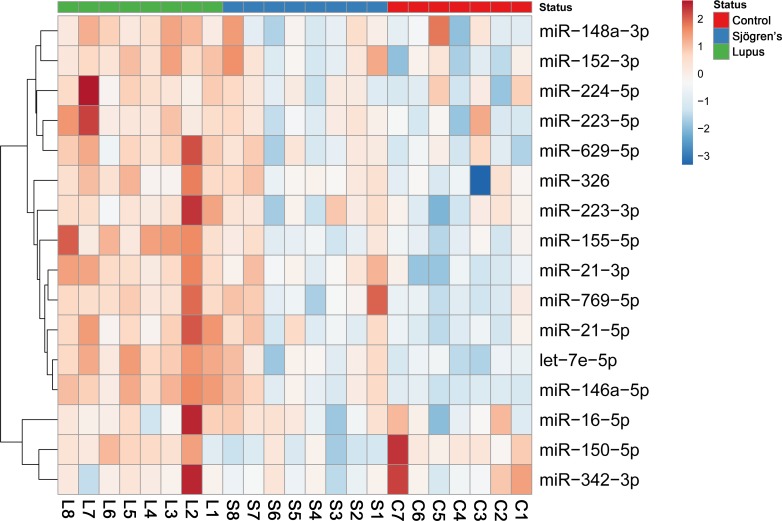
Expression profile of certain microRNAs in study individuals. Heatmap was generated using the expression data of 16 miRNAs potentially contribute to autoimmune disorders. The rows represent the individual miRNAs and columns represent our study individuals. Elevated expression level was signed by red colour.

Fig 4 shows the heat map representation [18] of the individual expression levels of certain miRNAs determined in the PBMCs of SLE and pSS patients and control subjects.

**Fig 5:**
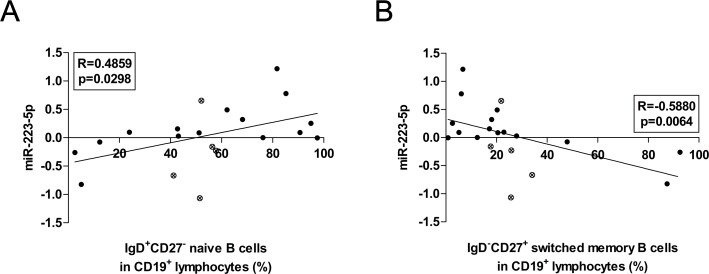
Associations between the expression levels of miR-223 and the percentages of peripheral lymphocyte subsets. The expression levels of miR-223-5p correlated positively with the proportions of naive B cells (A) and showed a negative correlation with switched memory B cell percentages (B). Each data point represents an individual subject. Black dots represents patients, while clear dots with an x show control subjects.

As a next step, we analysed the associations between the expression levels of miRNAs and the measured B lymphocyte ratios within PBMCs. The miR-223-5p showed a positive association with naive B cell percentages (R = 0.4859, p = 0.0298), and a negative correlation with switched memory B cell ratios (R = - 0.5880, p = 0.0064) (Fig 5A and 5B).

**Fig 6:**
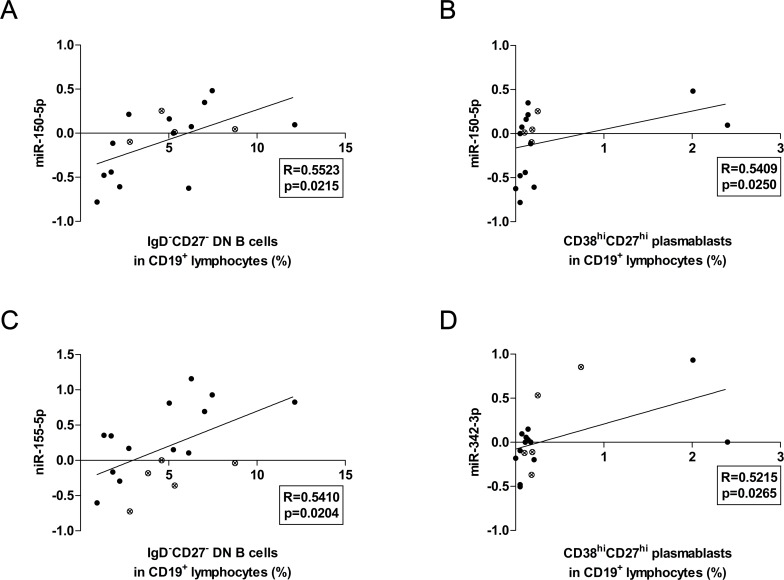
Associations between the expression levels of certain microRNAs and the percentages of peripheral lymphocyte subsets. The expression of miR-150-5p correlated positively with the ratios of both DN B cells (A) and plasmablasts (B), while other microRNAs (miRNAs), namely miR-155-5p (C) and miR-342-3p (D) showed positive associations with DN B cell and plasmablast ratios, respectively. Each data point represents an individual subject. Black dots represents patients, while clear dots with an x show control subjects.

The miR-150-5p expression levels showed significant positive correlations with the percentages of DN B cells (R = 0.5523, p = 0.0215) and plasmablasts (R = 0.5409, p = 0.0250) (Fig 6A and 6B). Furthermore, DN B cell ratios also showed significant positive correlations with the expression levels of miR-155-5p (R = 0.5410, p = 0.0204) (Fig 6C). Additionally, we revealed positive correlation between miR-342-3p expression levels and percentages of plasmablasts, as well (R = 0.5215, p = 0.0265) (Fig 6D).

## Discussion

In our study, we revealed moderated alterations in the expression patterns of miRNAs in patients with pSS. On the contrary, lupus patients showed more pronounced changes in miRNA expression profile, compared to the results of healthy subjects. Furthermore, the over-expressed miRNAs in pSS showed elevated expression levels in SLE, as well; although the decreased expression of miR-150 was observed in pSS patients only.

We compared the expression profiles of pSS and SLE patients in order to get a better insight into the disease-specific changes in the expression of different miRNAs. In total, 25 miRNAs were over-expressed in both SLE and pSS, including miR-146a, miR-16 and miR-21. Consequently, the observed alterations of these miRNAs could be part of the immunological processes developing in both investigated diseases. The over-expression of miR-146a/b is induced by LPS-mediated inflammatory responses and leads to translational repression of its target genes interleukin-1 receptor-associated kinase (IRAK) 1 and tumor necrosis factors receptor associated factor (TRAF) 6, thus serving as a negative feed-back for immune activation [19]. In T and B cells, group of miRNAs including miR-21, miR-146a, miR-155 and others might correlate with epigenetic modifications, support abnormal cytosine release, differentiation of cell subsets, B cell hyperactivity and autoantibody production [20]. Our workgroup previously reported the over-expression of miRNA-146 in pSS patients compared to controls [21]. Regarding SLE, a former study reported the under-expression of miR-146a in the PBMCs of Chinese SLE patients [22]. Nevertheless, our present study revealed the over-expression of miR-146a in PBMCs of our SLE patients. This variance between European and Chinese SLE patients could be explained by the difference in genetic background as well as various external factors, such as dietary habits, exposure to different infectious agents and other environmental elements, which may have effects on miRNAs expression [23,24]. The miR-16 is one of the critical regulators of TLR-mediated immune responses, and it can promote NF-kB regulated transactivation of the interleukin (IL)-8 gene by the suppression of silencing mediator for retinoid and thyroid hormone receptor (SMRT) [25]. MiR-16 was found to be up-regulated in patients with RA [9, 26], and based on our observations and considering the role of IL-8 in SLE, the miR-16 could play an important role in lupus as well. MiR-21 is regarded to be one of the DNA methylation associated miRNAs. It shows higher expression level in PBMCs of SLE patients compared to healthy controls [27]. MiR-21 has a pluripotent role in serving to link distinct lymphocyte signaling pathways and promote B and T cell activation in lupus. Overall, the elevated miR-21 expression observed in our SLE and pSS patients could be a part of autoimmune machinery, but presumably the consequence of an initial trigger, and not the reason.

In the present study, we found several over-expressed miRNAs in SLE, which levels were not changed in pSS. Hereby, we discuss the significance of miR-148a-3p, miR-152, miR-155, miR-223, miR-224, miR-326 and miR-342 in details. Over-expression of miR-148a impaired B cell tolerance by promoting the survival of immature B cells after engagement of the B cell antigen receptor by suppressing the expression of the autoimmune suppressor Gadd45α, the tumor suppressor PTEN and the pro-apoptotic protein Bim [28]. In our present study, we reported the over-expression of miR-148a in SLE; however, we also observed the same over-expression in pSS patients suffering from EGMs. Taken these together, the enhanced expression of this miRNA seems to be associated with systemic autoimmune processes, rather than a specific autoimmune disorder. It was also found that three members of miR-148 family, including miR-148a, miR-148b and miR-152, are negative regulators of the innate response and Ag-presenting capacity of DCs [29]. In the present study, the expression levels of both miR-148a-3p and miR-152 were elevated in the PBMCs of SLE patients. Beside the aforementioned miRNAs, miR-155 was also elevated in lupus, which is regarded as a central modulator of immune responses. Activated B and T cells show increased miR-155 expression, the same goes for macrophages and dendritic cells as well. MiR-155 is crucial for proper lymphocyte development and maturation and has been shown to be required for antibody production. Vigorito et al. reported that B cells lacking miR-155 generate reduced extrafollicular and germinal center responses and fail to produce high-affinity IgG1 antibodies, and that the transcription factor Pu.1 is a direct target of miR-155-mediated inhibition [30]. In autoimmune disorders such as rheumatoid arthritis, systemic sclerosis or psoriasis, miR-155 showed higher expression in patients' PBMCs [11]. In the present study, we observed enhanced expression of miR-155 in the PBMCs of our adult SLE patients, which was associated with the increased peripheral DN B cell percentages in lupus. A previous report proposed that the presence of these cells could be the result of an extrafollicular differentiation process in secondary lymphoid organs. Their activation does not require T cell interaction. Furthermore, due to the expression of CXCR3, after receiving activation signals, DN B cells could migrate to inflamed tissues [31,32]. Taken together, our observations underline the importance of miR-155 in not only germinal center but also extrafollicular responses in SLE. We observed elevated miR-223 expression levels in SLE. The present data on this miRNA is controversial, since it was reported that miR-223 is down-regulated in SLE patients with active nephritis in Denmark population [33]; while on the contrary, up-regulation was shown in SLE patients of Asian population [9]. In our study, we revealed the over-expression of both miR-223-3p and miR-223-5p in our SLE group; additionally, we demonstrated positive associations with naive B cells, and negative association with switched memory B cells. These associations are supported by the recent findings showing that the expression of miR-223 in naive B cells blocks the differentiation of naive B cells into GC B cells by repressing LIM domain only 2 (LMO2) and MYB Proto-Oncogene Like 1 (MYBL1). Conversely, a few highly up-regulated miRNAs in naive B cells, such as miR-223, miR-150 and miR-342-5p, were also down-regulated in activated peripheral blood B cells, suggesting that they regulate B cell activation [34]. Regarding miR-224, its over-expression facilitates activation-induced cell death in Jurkat cells. Enhanced expression of miR-224 accelerates T cell activation-induced cell death by suppressing apoptosis inhibitor (API)5 expression and associated with LN by enhancing STAT-1 expression in patients with SLE [35]. Our observation on over-expressed miR-224 in PBMCs underlines its significance in SLE pathogenesis. Additionally, we found elevated expression levels of miR-326, which was reported to be over-expressed in PBMCs from type 1 diabetic (T1D) patients with ongoing islet autoimmunity [36], however, its significance in lupus development needs to be further clarification. The miR-342-5p, similar to miR-491-5p, has predicted binding sites within the 3′UTR of three genes involved in Wnt signaling [Transcription Factor 7 (TCF7), musashi RNA-binding protein 1 (MSI1), and paired box 5 (PAX5)]. A recent study indicated that these miRNAs also participate in the regulation of TCF7, MSI1, and PAX5 genes. PAX5 upregulates lymphoid enhancer-binding factor 1 (LEF1) (TCF7-related protein, regulator of Wnt signaling) and interacts directly with LEF1 in B cells [37]. In our study, we observed the significant elevation in both miR-342-5p and miR-491-5p expression; moreover, miR-342-5p showed a strong positive correlation with plasmablast ratios in SLE. This is an important result, since PAX5 is one of the key transcription factors for plasma cell differentiation.

We observed the down-regulation of miR-150-5p, which is a novel and unique finding in pSS. Former studies showed that miR-150-5p might emerge as a master regulator of gene expression during the immune cells differentiation and immune response process. It plays an important role in the inhibition of B cell activation and differentiation, and its regulation ability in immune cellular process might contribute to the host defence against invading pathogens. Dysregulated expression of miR-150-5p in immune cells could result in autoimmune diseases [38]. MiR-150-5p was reported to be over-expressed in autoimmune pancreatitis compared to chronic pancreatitis, pancreatic cancer and healthy controls [39]. Based on our observations miR-150-5p is down-regulated in pSS, on the contrary, in SLE patients, miR-150-5p expression levels were not decreased but elevated, albeit not significantly. We also revealed that miR-150-5p expression levels associated positively with the percentages of DN B cells and plasmablasts. Our results are in accordance with the recently reported observations, namely, DN B cell percentages are lower in pSS, but higher in SLE, while plasmablast ratios are increased in SLE but not in pSS [17]. Based on these important differences, we assume that the under-expression of miR-150-5p potentially contributes to the differentiation and activation of B cells leading to the development of specific autoimmune processes in pSS.

In the present study, we not only depict the alterations in miRNA expression profiles in SLE and primary Sjögren’s syndrome, but we were the first to compare the changes in miRNA expression profiles between the two autoimmune diseases at the same time. However, we also have to mention some limitations of this study. SLE and pSS are clinically heterogeneous diseases and we worked with a relatively small sample size; therefore, our results should be confirmed in an independent set of patients. Some of the aforementioned miRNAs may be regarded as novel biomarkers for the investigated autoimmune disorders; however, functional experimental studies are also required to verify and establish the causal association between the aberrantly expressed miRNAs and the development of SLE and pSS.

In conclusion, the observed differences in miRNA expression profiles in Sjögren’s syndrome and systemic lupus erythematosus, and the better understanding of the immune regulatory mechanisms of the relevant miRNAs may help to elucidate the pathogenesis of the diseases. Certain miRNAs, as potential biomarkers, may not only help in the early diagnosis and prediction of prognosis, but also could assist in identifying potential targets for therapeutic interventions in the future.
